# Pedicle Screw Pseudofracture on Computed Tomography Secondary to Metal Artifact Reduction

**DOI:** 10.3390/diagnostics14010108

**Published:** 2024-01-03

**Authors:** Shuliang Ge, Naresh Kumar, James Thomas Patrick Decourcy Hallinan

**Affiliations:** 1 Department of Diagnostic Imaging, National University Hospital, 5 Lower Kent Ridge Road, Singapore 119074, Singapore; 2 University Spine Centre, Department of Orthopaedic Surgery, National University Health System, 1E, Lower Kent Ridge Road, Singapore 119228, Singapore; 3 Department of Diagnostic Radiology, Yong Loo Lin School of Medicine, National University of Singapore, 10 Medical Drive, Singapore 117597, Singapore

**Keywords:** artifacts, humans, prostheses and implants, spine surgery, radiographic image interpretation, computer-assisted methods, tomography, X-ray computed methods

## Abstract

Metal artifact reduction (MAR) algorithms are commonly used in computed tomography (CT) scans where metal implants are involved. However, MAR algorithms also have the potential to create new artifacts in reconstructed images. We present a case of a screw pseudofracture due to MAR on CT.

The patient was a 52-year-old female with a past medical history of type 2 diabetes mellitus, hyperlipidemia, and nonalcoholic steatohepatitis with liver cirrhosis. She had a surgical history of laparoscopic sleeve gastrectomy for obesity. The patient underwent O-arm-guided L3-L5 spinal instrumentation using a Globus Revere system, L3-L5 intertransverse grafting for L4-L5 degenerative spondylolisthesis, and L5-S1 degenerative disc disease. Postoperatively, the patient had an anteriorly extruded bone graft; hence, routine outpatient follow-up CT scans were performed to monitor this patient.

On one of the follow-up CT scans performed 3 months after surgery, there was a linear dark band across the right L3 pedicle screw that was visible on both axial and sagittal reconstructions performed with MAR (Figure 1). Otherwise, there was no evidence of implant loosening or peri-implant fracture. The spinal alignment was stable compared to the previous postoperative CT scan. 

The linear dark band across the right L3 pedicle screw was initially interpreted as a screw fracture. Subsequently, the patient was recalled for early assessment in the spine clinic and attended a few days later. She did not report any episodes of trauma post surgery, new pain, or symptoms. Radiographs were also acquired at this appointment and did not reveal any disruption of the right L3 pedicle screw (Figure 2). The spine surgeon requested reassessment of the CT images by the radiologist, and further review of the native non-MAR images showed that the right L3 pedicle screw was intact (Figure 1c). The findings on the MAR images were deemed to be secondary to the artifact introduced in the reconstruction process.

Metal implants are known to cause artifacts due to photon starvation and beam hardening effects [1]. These artifacts can limit the assessment of implant loosening, peri-implant fractures, and adjacent soft tissue. Commercial MAR algorithms have been developed by various CT vendors, often using projection–interpolation methods and iterative processes [2]. Dual-energy CT can also help increase the image quality when metal implants are involved. The main application of these MAR algorithms is to improve the visualization of peri-implant structures, hence allowing for the better assessment of implant loosening, fractures, and soft tissue changes [3,4].

While MAR algorithms on CT can improve the visualization of peri-implant structures, they also have the potential to create new artifacts such as bright and dark streaks, as well as the disappearance of metallic implants and underestimation of implant size [5,6,7,8,9,10]. These new artifacts are due to several reasons (Table 1).

In the recent literature, most new artifacts introduced by MAR are not as dramatic as mimicking an implant failure. However, there is one previously reported case of an MAR artifact resulting in the appearance of a pedicle screw pseudofracture [12].

The type of metal implant material may also affect the outcome of MAR algorithms. Previous phantom studies have shown that MAR improves artifacts and image quality with cobalt-chrome and stainless-steel implants. Conversely, titanium implants do not cause severe artifacts before MAR, but new or more severe artifacts have been observed after MAR, leading to a similar or decreased image quality [13]. In practice, it is difficult to immediately determine the implant material during scan interpretation, and some patients may also have implants made of a mixture of different materials. 

Our patient underwent imaging on a dual-energy General Electric (GE) Revolution CT scanner using Gemstone Spectral Imaging (GSI) with and without reconstruction via the MAR algorithm failure. The artifacts in our case were due to segmentation and/or interpolation. In other words, the software may have failed to identify the pedicle screw correctly, erroneously recognized part of the screw as corrupted projection data, and removed it.

These new artifacts introduced by MAR are not unique to a specific MAR algorithm or CT scanner and have been demonstrated across different machines from various vendors. We noted in one previous study comparing O-MAR images with high-keV monoenergetic images that O-MAR was responsible for more of these new artifacts [14]; however, the literature specific to comparing and resolving new artifacts is limited. The best way to avoid this pitfall is still to have the original images without MAR available for review.

In summary, while CT MAR algorithms can provide better visualization of peri-implant structures and improve diagnostic confidence, these algorithms are also known to create new artifacts which in our case mimicked a pedicle screw fracture. Readers should be cognizant of this phenomenon and MAR images should always be interpreted together with non-MAR images.

## Figures and Tables

**Figure 1:**
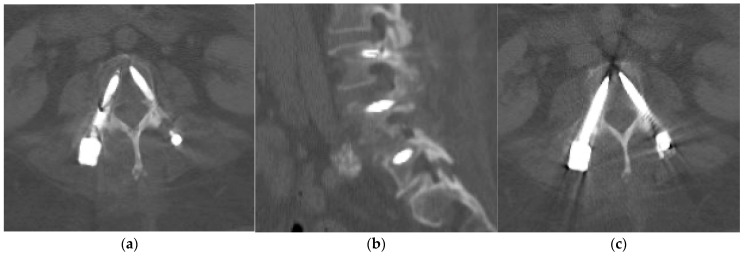
Axial (**a**) and sagittal (**b**) CT bone reconstructions with MAR showing a linear dark band across the mid-section of the right L3 pedicle screw. Axial CT bone reconstruction without MAR (**c**) at the same L3 level showing intact screws.

**Figure 2:**
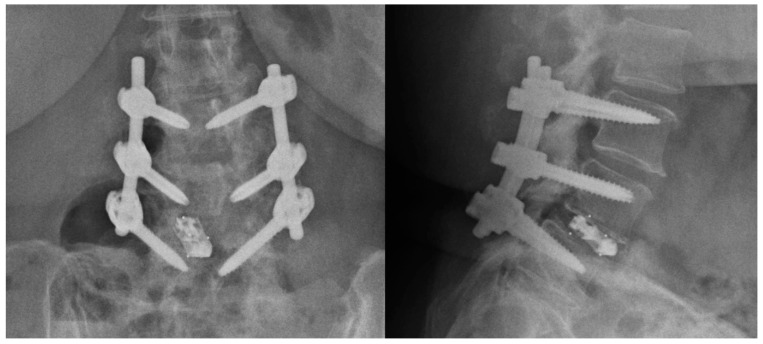
Anterior–posterior and lateral radiographs of the implants were taken a few days after the CT scan and showed intact pedicle screws.

**Table 1:** Proposed reasons for new artifacts observed with CT MAR algorithms [2,6,9,11].

Reasons	Details
Incorrect metal segmentation	The first step of MAR algorithms is to segment the high-density structures such as metal implants. This can be performed using a Hounsfield unit threshold. The segmentation is then used for subsequent projection–interpolation and iterative processes.
Errors in projection data	Once the metal is segmented, the algorithm tries to identify the corrupted projection data corresponding to the segmented metal. The corrupted data can then be removed and interpolated with estimations of uncorrupted data. The process is then iterated multiple times. There can be errors in this process, mistaking metal or surrounding tissue as corrupted data.
Artifacts beyond photon starvation	While MAR algorithms attempt to reduce the effects of photon starvation and beam hardening, other artifacts such as metal scattering and partial voluming exist and are not fully corrected by MAR.

## Data Availability

The data in this report are available from the corresponding authors upon request.
